# Patterns of Clinical Trial Enrollment for Pediatric Patients With Hepatoblastoma and Wilms Tumor: A Report From the Children's Oncology Group

**DOI:** 10.1002/cam4.70692

**Published:** 2025-03-27

**Authors:** Pablo S. Monterroso, Sarah Lucht, Jeannette M. Sample, Angela D. Trobaugh‐Lotrario, Helen M. Parsons, Lucie M. Turcotte, David Van Riper, Jenny N. Poynter, Erin L. Marcotte

**Affiliations:** ^1^ Division of Epidemiology and Clinical Research, Department of Pediatrics University of Minnesota Minneapolis Minnesota USA; ^2^ Environmental Epidemiology Group, Institute for Occupational, Social and Environmental Medicine, Centre for Health and Society Medical Faculty and University Hospital Düsseldorf, Heinrich Heine University Düsseldorf Düsseldorf Germany; ^3^ Department of Pediatric Hematology/Oncology Providence Sacred Heart Children's Hospital Spokane Washington USA; ^4^ Division of Health Policy and Management University of Minnesota Minneapolis Minnesota USA; ^5^ Masonic Cancer Center University of Minnesota Minneapolis Minnesota USA; ^6^ Division of Hematology and Oncology, Department of Pediatrics University of Minnesota Minneapolis Minnesota USA; ^7^ Minnesota Population Center University of Minnesota Minneapolis Minnesota USA

**Keywords:** childhood cancer, clinical trials, pediatric, race and ethnicity, socioeconomic status

## Abstract

**Background:**

Published childhood cancer studies have observed differences in therapeutic trial enrollment by race, ethnicity, socioeconomic status (SES), and age at diagnosis. Our study investigates patterns of enrollment for pediatric oncology clinical trials.

**Methods:**

We analyzed differences in Children's Oncology Group clinical trial enrollment in a cohort of pediatric patients with hepatoblastoma (*n* = 212) and Wilms tumor (*n* = 1107). Relative risks (RRs) and 95% confidence intervals (95% CIs) were estimated for trial enrollment by patient characteristics. Odds ratios (ORs) and 95% CIs were estimated to examine associations between characteristics and three outcomes (therapeutic trial [referent], exclusively non‐therapeutic study, no trial or study). Statistical significance tests were two‐sided.

**Results:**

Approximately half of all cases enrolled in therapeutic trials for both tumor types (Wilms: 48%; hepatoblastoma: 51%). For Wilms tumor, patients diagnosed at ages 3–5 years were more likely to enroll compared to those diagnosed at age < 1 year (RR = 1.06; 95% CI = 1.01, 1.13) and had lower odds of joining exclusively a non‐therapeutic study compared to those diagnosed at age < 1 years (OR = 0.63; 95% CI = 0.44, 0.90). There was no association between race, ethnicity, or SES and enrollment. For hepatoblastoma, no variables indicated any statistically significant difference in enrollment.

**Conclusions:**

Few differences in clinical trial enrollment were observed during periods when trials were available for all risk groups, a promising sign of equity in pediatric oncology clinical trial recruitment. Among Wilms tumor cases, differences in enrollment were observed for age at diagnosis, a potential proxy for disease acuity, which may influence decision making.

## Graphical abstract

Our study examines disparities in enrollment for pediatric oncology clinical trials, focusing on pediatric patients with hepatoblastoma and Wilms tumor. We found few disparities in trial enrollment, though young age at diagnosis was a statistically significant predictor for Wilms tumor cases.

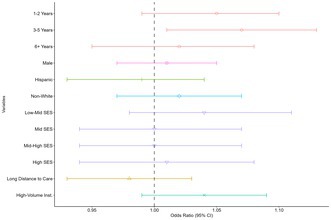

## 1 Introduction

In 2024, an estimated 14,910 children and adolescents aged 0–19 years were diagnosed with cancer in the United States [1]. While childhood cancer is rare, incidence rates have been increasing since 1975 by almost 1% per year through 2019, with notable disparities present [1, 2]. A recent analysis of trends from 2000 to 2019 found increasing incidence rates among lower socioeconomic status (SES) populations, while incidence remained relatively stable among the highest SES group [2]. Since the mid‐1970s, there has been a marked improvement in 5‐year survival rates, which increased from 58% to 85% for all cancers combined, although there is substantial variation in survival by cancer type and demographics [1, 3, 4]. The decrease in mortality is attributable to refined risk stratification and therapeutic advances due to the success of collaborative clinical trials conducted by multi‐institutional organizations such as the Children's Oncology Group (COG) [5, 6]. While prior work has suggested that clinical trial enrollment improves outcomes, not all groups are represented equally [7, 8, 9, 10, 11, 12]. Differences in enrollment by race, ethnicity, SES, and age at diagnosis have been well described in adult populations [13, 14, 15, 16, 17, 18]. The pediatric population is understudied in this area; the few publications on trial enrollment report that individuals from racial/ethnic minority populations are underrepresented in therapeutic clinical trials [19, 20, 21, 22, 23, 24]. The most common reason for non‐enrollment is lack of an available trial, which may be a limiting factor in attempts to categorize differences where newly diagnosed cases may not have a trial available for enrollment [25].

Minimizing gaps in enrollment is crucial to ensuring the generalizability of results and health equity. Characterizing the population of patients enrolled in trials and identifying barriers to participation is a key step in this process. There are no published reports on clinical trial enrollment differences among pediatric patients with hepatoblastoma or nephroblastoma (Wilms tumor). Hepatoblastoma incidence is increasing overall and most rapidly for non‐Hispanic Black and Hispanic children and adolescents [2], and incidence rates are highest among Hispanic children [26]. Unlike many other tumor types, Black children have the highest incidence rate of Wilms tumor [26]. Thus, these are important patient populations to examine with regard to therapeutic clinical trial enrollment for these two cancer types.

To facilitate research on childhood cancer etiology and outcomes, the COG established the Childhood Cancer Research Network (CCRN) in December 2007 [27]. The CCRN is a case recruitment protocol across the United States (U.S.) and Canada, which includes consent to contact for future research. Enrollment onto the COG's CCRN is optional, and it is reported that CCRN represents approximately 36% of expected newly diagnosed childhood cancer cases age < 19 years, based on estimates derived from the National Cancer Institute's Surveillance, Epidemiology, and End Results (SEER) Program [28]. Examination of the entire CCRN cohort revealed demographic differences in observed‐to‐expected enrollment rates, whereby non‐Hispanic White patients and patients diagnosed between 1 and 9 years of age had the highest rates [28]. Notably, 60% of the expected number of Wilms tumor patients and 47% of hepatoblastoma patients enrolled in the CCRN.

Here, we aim to identify whether there are differences by age, sex, race/ethnicity, area‐based SES, institution type, and distance to care in COG clinical trial enrollment for pediatric patients with hepatoblastoma and Wilms tumor. To address previous gaps and limitations in the literature, we employ a multicenter analysis focused on patients diagnosed during a period when disease‐specific clinical trials for all risk groups were open.

## 2 Methods

### 2.1 Study Population

Clinical trial enrollment data were obtained from the COG Childhood Cancer Research Network (CCRN). The CCRN protocol created a research registry from over 200 COG institutions across the United States and Canada, and recruited children diagnosed with all tumor types, regardless of clinical trial enrollment, from December 2007 to December 2017 (*n* = 57,748) [27, 28].

In this analysis, cases were restricted to pediatric patients diagnosed with hepatoblastoma or Wilms tumor during the period when disease‐specific clinical trials for all risk groups were open, theoretically making the majority of newly diagnosed cases eligible for participation. All clinical trials were multicenter Phase III frontline therapeutic trials. The COG protocol AHEP0731 was utilized for hepatoblastoma case finding during the period when all strata were open (9/14/2009–03/12/2012). More information is available on clinicaltrials.gov, identifier NCT00980460. For Wilms tumor, the COG protocols AREN0532, AREN0533, AREN0534, and AREN0321 were utilized for case finding during the period when all trials, comprising all risk groups, were open (7/13/2009–05/24/2013). More information is available on clinicaltrials.gov, identifiers NCT00352534, NCT00379340, NCT00945009, NCT00335556. Non‐therapeutic studies included tissue specimen biology banking protocols, supportive care studies, and long‐term follow‐up studies (Table S1).

Race/ethnicity, sex, diagnosis, age at diagnosis, and other patient characteristics were obtained as reported in CCRN [28]. CCRN registration also included the provision of an address that was current at the time of registration. The Yost Index, developed by Yost et al. 2001, measures SES at the Census block group level utilizing seven key inputs related to educational level, income, housing, and employment [29]. This was computed from exact addresses through geocoding. Distance to care was calculated based on the direct distance from each patient's exact address to the designated care institution (point to point), with this distance metric also derived from the geocoding of patient locations. Addresses that were resolved to an address point or street address were matched to their census block. Addresses that were only geocoded to a lower resolution (street name, zip code, city, or unmatched) were not able to be matched to a census block or tract and were considered a poor geocoding match.

Cases with missing data on age, sex, race, ethnicity, year of diagnosis, participation in a therapeutic trial, Yost Index (a measure of neighborhood SES), and distance to care were excluded (hepatoblastoma *n* = 3; Wilms tumor *n* = 177). Those with a poor geocoding match were also excluded (hepatoblastoma *n* = 3; Wilms tumor *n* = 54). Cases were further excluded if St. Jude Children's Research Hospital served as their treating institution (hepatoblastoma *n* = 4; Wilms tumor *n* = 28). This exclusion was implemented due to likely misclassification of the distance to care variable. Upon inspection of the distribution of distance to care, we found that there were a large number of cases in the greater Baton Rouge and Shreveport, LA, Peoria, IL, and Johnson City, TN, areas with > 250 miles distance to their treatment institution, although in many instances there were COG institutions within 100 miles or less of the geocoded address. We examined the listed treatment institutions of these cases and found that > 95% were St. Jude in Memphis, TN. Although it is likely that these individuals were treated at the St. Jude affiliate hospitals in their regions, we are not able to make this determination without additional data. The consequential impact on the distance to care variable prompted their exclusion.

### 2.2 Statistical Analysis

For the binary outcome of enrollment in a therapeutic clinical trial (yes/no), we ran a Poisson model with robust variance to estimate relative risks (RRs). This model was adjusted for age at diagnosis, sex, ethnicity (Hispanic/non‐Hispanic), race (White/non‐White), long distance to care (> 60 miles from care institution), high‐volume institution (top quartile for the CCRN dataset for that tumor type), and Yost Index (quintiles). Exponentiated coefficients were used to present relative risks. 95% confidence intervals (95% CIs) were fitted via robust variance.

For the multiple category outcome (therapeutic trial, exclusively non‐therapeutic study, no trial or study), we ran a multinomial logistic regression model with therapeutic trial enrollment as the reference. CCRN enrollment was not considered within the category of “no trial or study” as all included patients were on the registry. Models included age at diagnosis, sex, ethnicity, race, long distance to care, high‐volume institution, and Yost Index. Estimates after exponentiating coefficients are presented as odds ratios (ORs). Demographic distributions of cases during the period when any strata were open, along with an expanded analysis, are available in Tables S2, S3, and Figures [Link], [Link], [Link], [Link], respectively. Tests of statistical significance were 2‐sided, and the threshold for statistical significance was set at an alpha of 0.05. All analyses were done using RStudio 2023.06.2.

## 3 Results

In general, the statistical estimates for associations between demographic or socio‐economic characteristics and the likelihood of therapeutic trial enrollment did not reveal strong effect estimates and generally lacked precision, indicating limited evidence of strong demographic predictors in trial participation.

### 3.1 Wilms Tumor

Of the 1107 patients diagnosed during the analyzed period, 534 (48%) enrolled in therapeutic trials, 489 (44%) enrolled exclusively in non‐therapeutic studies, and 84 (8%) did not participate in any protocols beyond CCRN. The majority of patients were White (75%). Demographic distributions were similar across trial enrollment status categories and overall, as presented in Table 1.

**TABLE 1:** Demographic characteristics of Wilms tumor patients analyzed.

		Trial enrollment				
Characteristic	*N*	Clinical trial, *N* = 534^a^	Non‐clinical trial, *N* = 489^a^	None, *N* = 84^a^	Overall, *N* = 1,107^a^	*p* ^b^
Sex	1107					0.6
Female		280 (52%)	259 (53%)	49 (58%)	588 (53%)	
Male		254 (48%)	230 (47%)	35 (42%)	519 (47%)	
Race	1107					
White		398 (75%)	374 (76%)	62 (74%)	834 (75%)	
Black		98 (18%)	81 (17%)	14 (17%)	193 (17%)	
Native American/Alaska native		3 (0.6%)	3 (0.6%)	0 (0%)	6 (0.5%)	
Asian/PI		8 (1.5%)	11 (2.2%)	2 (2.4%)	21 (1.9%)	
Other		27 (5.1%)	20 (4.1%)	6 (7.1%)	53 (4.8%)	
Hispanic, yes	1107	83 (16%)	77 (16%)	19 (23%)	179 (16%)	0.2
Age at diagnosis (years)	1107					0.13
< 1		126 (24%)	146 (30%)	18 (21%)	290 (26%)	
1–2		187 (35%)	159 (33%)	27 (32%)	373 (34%)	
3–5		132 (25%)	97 (20%)	19 (23%)	248 (22%)	
6+		89 (17%)	87 (18%)	20 (24%)	196 (18%)	
Yost index (quintiles)	1107					0.5
Low SES		105 (20%)	108 (22%)	11 (13%)	224 (20%)	
Low‐mid SES		109 (20%)	83 (17%)	15 (18%)	207 (19%)	
Mid SES		101 (19%)	93 (19%)	22 (26%)	216 (20%)	
Mid‐high SES		107 (20%)	103 (21%)	19 (23%)	229 (21%)	
High SES		112 (21%)	102 (21%)	17 (20%)	231 (21%)	
Distance to care, per 50 km	1107	0.58 (0.28, 1.43)	0.65 (0.27, 1.49)	0.59 (0.27, 2.00)	0.62 (0.28, 1.51)	> 0.9
High volume institution	1107	395 (74%)	337 (69%)	61 (73%)	793 (72%)	0.2

^a^ *n* (%); Median (IQR).

^b^ Pearson's Chi‐squared test; Kruskal–Wallis rank sum test.

**FIGURE 1:**
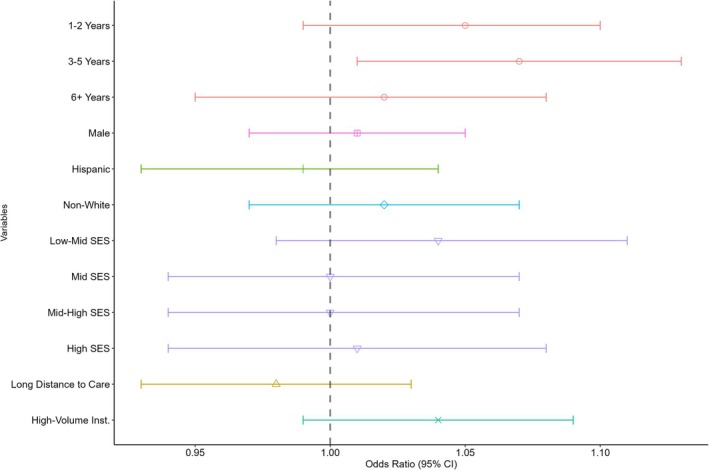
Relative risks of therapeutic trial enrollment by patient characteristics for Wilms tumor patients.

When examining factors associated with therapeutic trial enrollment (Figure 1), patients aged 3–5 years at the time of diagnosis were more likely to enroll in therapeutic trials compared to those aged < 1 year at diagnosis (RR = 1.06; 95% CI = 1.01, 1.13). No other variables indicated statistically significant changes in the likelihood of enrollment.

**FIGURE 2:**
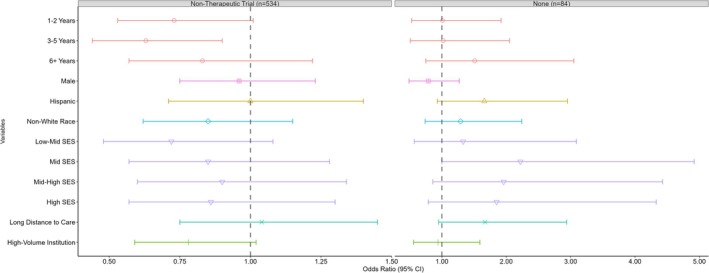
Odds ratios of exclusively non‐therapeutic study or no trial enrollment for Wilms tumor patients (therapeutic trial enrollment as reference group).

In multinomial analysis (Figure 2), patients aged 3–5 years at diagnosis were less likely to enroll exclusively in non‐therapeutic studies as compared to therapeutic trials when compared to those aged < 1 year at diagnosis (OR = 0.63; 95% CI = 0.44, 0.90). Although patients aged 1–2 years at diagnosis (OR = 0.73; 95% CI = 0.53, 1.01) and those living in a neighborhood in the low‐mid SES quintile (OR = 0.72; 95% CI = 0.48, 1.08) showed decreased odds of enrolling in exclusively non‐therapeutic studies when compared to those aged < 1 year at diagnosis and in the low SES quintile, these associations were not statistically significant.

Patients from the mid SES group showed higher odds of enrolling in therapeutic trials versus no trial compared to those from the low SES group (OR = 2.22; 95% CI = 1.00, 4.92). The only attribute associated with a greater likelihood of enrolling in no trial compared to a therapeutic trial was male sex, although the confidence interval included the null.

### 3.2 Hepatoblastoma

Of the 212 patients diagnosed during the analyzed period, 109 (51%) enrolled in therapeutic trials, 26 (12%) enrolled exclusively in non‐therapeutic studies, and 77 (36%) did not participate in any protocols other than CCRN. The majority of patients were White (72%), male (62%), and aged < 1 year at diagnosis (59%). Demographic characteristics varied by trial enrollment status based on race and year of diagnosis, while other characteristics were consistent across groups, as shown in Table 2.

**TABLE 2:** Demographic characteristics of hepatoblastoma patients analyzed.

		Trial enrollment				
Characteristic	N	Clinical trial, *N* = 109^a^	Non‐clinical trial, *N* = 26^a^	None, *N* = 77^a^	Overall, *N* = 212^a^	*p* ^b^
Sex	212					> 0.9
Female		40 (37%)	10 (38%)	30 (39%)	80 (38%)	
Male		69 (63%)	16 (62%)	47 (61%)	132 (62%)	
Race	212					0.4
White		75 (69%)	21 (81%)	57 (74%)	153 (72%)	
Black		13 (12%)	0 (0%)	8 (10%)	21 (9.9%)	
Native American/Alaska native		0 (0%)	0 (0%)	0 (0%)	0 (0%)	
Asian/PI		11 (10%)	2 (7.7%)	3 (3.9%)	16 (7.5%)	
Other		10 (9.2%)	3 (12%)	9 (12%)	22 (10%)	
Hispanic, yes	212	28 (26%)	7 (27%)	17 (22%)	52 (25%)	0.8
Age at diagnosis (years)	212					0.2
< 1		70 (64%)	19 (73%)	36 (47%)	125 (59%)	
1–2		24 (22%)	5 (19%)	25 (32%)	54 (25%)	
3–5		6 (5.5%)	1 (3.8%)	8 (10%)	15 (7.1%)	
6+		9 (8.3%)	1 (3.8%)	8 (10%)	18 (8.5%)	
Yost index (quintiles)	212					
Low SES		25 (23%)	4 (15%)	18 (23%)	47 (22%)	
Low‐mid SES		15 (14%)	5 (19%)	12 (16%)	32 (15%)	
Mid SES		23 (21%)	3 (12%)	17 (22%)	43 (20%)	
Mid‐high SES		26 (24%)	5 (19%)	17 (22%)	48 (23%)	
High SES		20 (18%)	9 (35%)	13 (17%)	42 (20%)	
Distance to care, per 50 km	212	0.87 (0.39, 1.93)	0.68 (0.32, 1.76)	0.66 (0.35, 1.76)	0.74 (0.37, 1.84)	0.7
High volume institution	212	54 (50%)	12 (46%)	39 (51%)	105 (50%)	> 0.9

^a^ *n* (%); Median (IQR).

^b^ Pearson's Chi‐squared test; Fisher's exact test; Kruskal–Wallis rank sum test.

**FIGURE 3:**
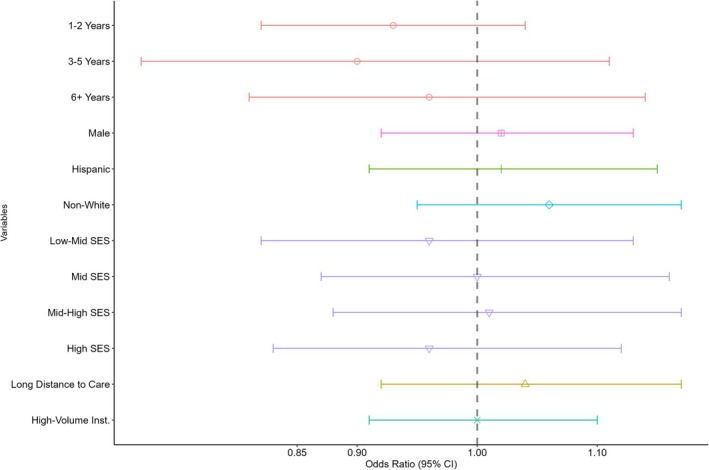
Relative risks of therapeutic trial enrollment by patient characteristics for hepatoblastoma patients.

No statistically significant associations between socio‐demographic characteristics and the likelihood of enrollment in a therapeutic trial were observed (Figure 3). No clear trends were observed by SES quintile or age group.

**FIGURE 4:**
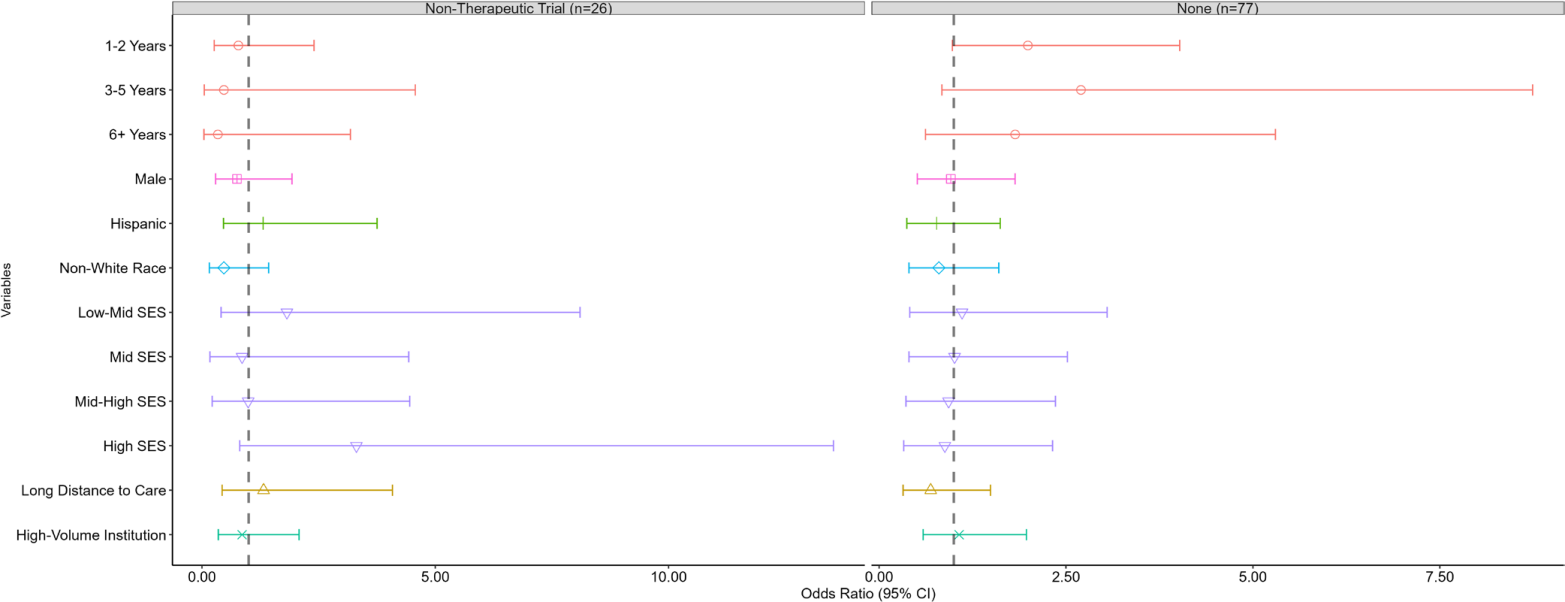
Odds ratios of exclusively non‐therapeutic study or no trial enrollment for Wilms tumor patients (therapeutic trial enrollment as reference group).

In the multinomial analysis (Figure 4), although patients living in high SES quintile neighborhoods showed higher odds of enrolling in therapeutic trials compared to exclusively non‐therapeutic studies, relative to patients living in low SES quintile neighborhoods (OR = 3.31; 95% CI = 0.81, 13.53), this association was not statistically significant. Similarly, increased age at diagnosis and being non‐White showed point estimates indicating decreased likelihoods of enrolling in therapeutic trials compared to non‐therapeutic studies, although these analyses lack statistical precision and the confidence intervals include the null.

Diagnosis at age 3–5 years showed an elevated point estimate for enrolling in therapeutic trials compared to no trial, relative to diagnosis at age < 1 year (OR = 2.70; 95% CI = 0.84, 8.74), but this difference did not reach statistical significance. Additionally, living more than 60 miles from the treatment institution was associated with a lower odds of enrolling in therapeutic trials compared to no trial (OR = 0.69; 95% CI = 0.32, 1.49), but this association was not statistically significant. Overall, no clear patterns were observed between SES quintile and type of trial enrollment compared to no enrollment.

## 4 Discussion

Understanding differences in clinical trial enrollment remains a pressing concern within the realm of pediatric oncology. Our study aimed to examine therapeutic trial enrollment within the context of pediatric patients with hepatoblastoma and Wilms tumor, representing the first analysis of trial enrollment patterns in these populations. Close to half of all cases in our cohort enrolled in a clinical trial for hepatoblastoma and Wilms tumor, respectively. Our findings indicate that age at diagnosis significantly influenced trial enrollment for Wilms tumor patients, with children diagnosed at ages 3–5 years showing a higher likelihood of enrollment compared to those diagnosed at age < 1 year.

The enrollment rates observed in our analysis are consistent with those reported previously for COG clinical trials [27, 28]. Our analysis did not reveal any statistically significant associations between race/ethnicity or SES and trial enrollment within our study cohort. While the lack of differences in enrollment is a promising sign for health equity within this study population, this stands in contrast to prior studies, where differences in enrollment by race/ethnicity and sex have been reported [19, 20, 21, 22, 23, 24]. It is important to note that differences in race/ethnicity and SES may still exist in other cancer types not covered by this analysis. The finding that ages 3–5 years at diagnosis are more likely to enroll on Wilms tumor trials compared to those aged < 1 year at diagnosis may be due to factors influencing parents' decision making regarding enrollment, such as perceived risk/benefit, how study team members communicate treatment options, child willingness to participate, and trust in the study team [30, 31].

Unequal enrollment in clinical trials is a barrier to achieving health equity, particularly given the association between trial participation and improved outcomes alongside more effective long‐term follow‐up [32]. Prior studies have observed that racial/ethnic minority populations often exhibit low enrollment rates in clinical trials [33]. We hypothesized that non‐White racial groups, Hispanic ethnic groups, and populations living in lower SES areas and at greater distances to care would be less likely to enroll in therapeutic clinical trials. Although we did not observe statistically significant differences in enrollment among these groups, it is possible that the lack of detailed clinical and eligibility data masked some associations. For instance, a recent analysis of hepatoblastoma cases observed that many were ineligible due to inadequate pulmonary function or hypoxia, as well as additional comorbidities such as poor performance status and inadequate renal function [34]. Indeed, it is known that both hepatoblastoma and Wilms tumor cases have high rates of congenital anomalies, including an overrepresentation of cardiac defects among hepatoblastoma cases [35]. This could be due to an increasing number of patients with trisomy 18 (who have an increased risk of congenital heart disease) surviving the neonatal/early childhood period recently and subsequently developing hepatoblastoma [36]. In addition, patients with very low birth weight, who are also known to have an increased risk of hepatoblastoma [37], often have comorbidities that include hypoxia, chronic lung disease, and chronic oxygen requirement; they are also thus ineligible for enrollment in trials. This could explain the decrease in enrollment in trials compared to patients with Wilms tumor. Due to a lack of clinical and other covariates, we were not able to assess ineligibility, and therefore non‐enrollment, due to these factors. Finally, although we did not observe differences in this analysis, it is crucial to acknowledge the presence of race/ethnicity and SES differences in clinical trial participation reported in the literature and to further understand barriers to participation among all populations.

Strengths of this study include its focus on underrepresented pediatric cancer types, the utilization of COG data including registry participants who did not participate in therapeutic trials, and its potential to guide future research efforts. This study represents the first analysis of hepatoblastoma and Wilms tumor trial enrollment differences. Our deliberate focus on patients diagnosed during the period when clinical trials for all risk groups were open ensures the majority of newly diagnosed cases were theoretically eligible for participation, thereby strengthening our analysis. This approach also helps mitigate the impact of a lack of available staging information.

While case counts were low, the entire CCRN cohort represents 36% of all newly diagnosed cases during this period and 50% of cases diagnosed between 1 and 4 years [27, 28]. Despite this, there were insufficient case counts to stratify by specific racial or specific groups. We are limited by having data on only CCRN enrollees, which potentially limits generalizability if the CCRN population is not representative of the entire populations of patients with Wilms tumor and hepatoblastoma. Prior work comparing the CCRN population to that of SEER suggests that 60% of Wilms tumor patients and 47% of hepatoblastoma patients enrolled on CCRN. Overall, there were some differences in observed to expected ratios by race/ethnicity, with non‐Hispanic White patients having the highest enrollment. Additionally, while our study focused specifically on clinical trial enrollment and did not identify differences by race or SES in this context, existing literature suggests that disparities in care may still exist for certain populations within pediatric oncology. For example, a recent study reported disparities in care for Black children with hepatoblastoma [38], suggesting that systemic inequities may affect health outcomes beyond clinical trial access.

Distance to care was calculated using the point‐to‐point method as opposed to using network analysis, potentially leaving out the effect of constraints such as traffic and road types. Our dataset lacked insurance information, limiting our ability to consider its impact. We were also limited in clinical data and could not assess ineligibility due to organ function or other factors, as noted above. There is sometimes lag time between when COG opens a study and when each institution opens the study, which may have affected the ability of some patients to enroll. Of note is the limited availability of clinical trials for hepatoblastoma patients during the study period. As a result, there were small case counts for this population. With only one trial accessible to this patient population, our study underscores the scarcity of trial opportunities for this rare cancer.

By exploring the intricacies of clinical trial enrollment differences, our study contributes to the ongoing efforts to enhance pediatric oncology care. Future research in this field should continue to explore differences in clinical trial enrollment among pediatric cancer patients. Leveraging innovative approaches, such as linking clinical trial data with cancer registries, as exemplified by Siegel et al., may provide novel insights into enrollment patterns [39]. It is crucial to identify and address specific barriers at both individual and systemic levels, which hinder equal access to clinical trials. This may involve examining the impact of factors such as health literacy, healthcare infrastructure, and access to healthcare on decision‐making processes related to pediatric clinical trial enrollment. Furthermore, interventions and strategies aimed at increasing awareness and participation in clinical trials among underrepresented populations should be developed and implemented. Addressing these differences is essential to working towards a future where all children have equal access to novel therapies, thereby improving their chances of successful outcomes and ultimately ensuring health equity in pediatric oncology.

## Author Contributions

**Pablo S. Monterroso:** data curation (equal), formal analysis (equal), writing – original draft (lead), writing – review and editing (equal). **Sarah Lucht:** data curation (equal), formal analysis (equal), writing – review and editing (equal). **Jeannette M. Sample:** data curation (equal), writing – review and editing (equal). **Angela D. Trobaugh‐Lotrario:** writing – review and editing (equal). **Helen M. Parsons:** writing – review and editing (equal). **Lucie M. Turcotte:** writing – review and editing (equal). **David Van Riper:** writing – review and editing (equal). **Jenny N. Poynter:** writing – review and editing (equal). **Erin L. Marcotte:** conceptualization (equal), methodology (equal), supervision (equal), writing – review and editing (equal).

## Disclosure

The content is solely the responsibility of the authors and does not necessarily represent the official views of the National Institutes of Health.

## Ethics Statement

This study was approved by the Institutional Review Board at the University of Minnesota prior to commencing this study. Written informed consent for participation in the Childhood Cancer Research Network registry was obtained by participating Children's Oncology Group institutions.

## Conflicts of Interest

The authors declare no conflicts of interest.

## Supporting information


Figure S1.

Figure S2.

Figure S3.

Figure S4.

Table S1.

## Data Availability

The data that support the findings of this study are available from the corresponding author upon reasonable request.
