# Supplementary figures and images for: Patterns of Clinical Trial Enrollment for Pediatric Patients With Hepatoblastoma and Wilms Tumor: A Report From the Children's Oncology Group
Source: Cancer Med. 2025 Mar 27;14(7):e70692. doi: 10.1002/cam4.70692 (PMC11950632; doi:10.1002/cam4.70692)

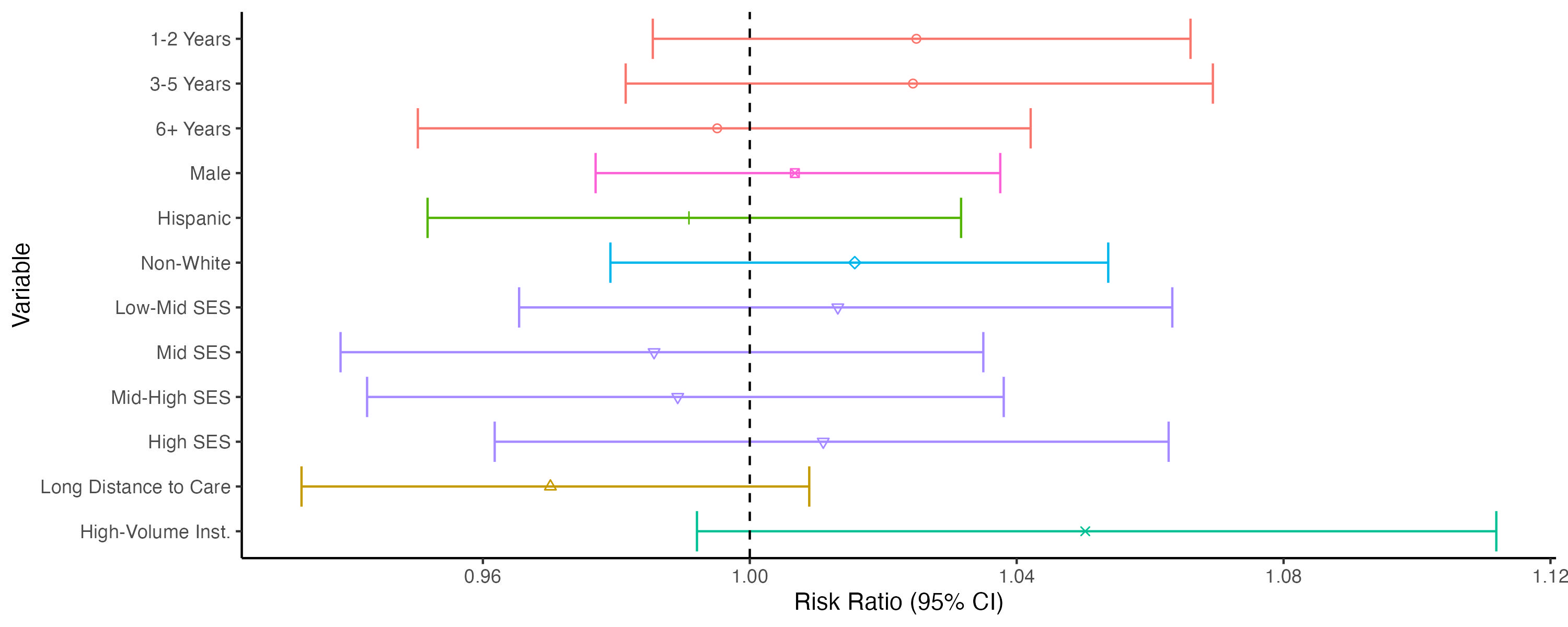

Supplement: Supplementary file 1 — Figure S1. [file CAM4-14-e70692-s005.jpeg]

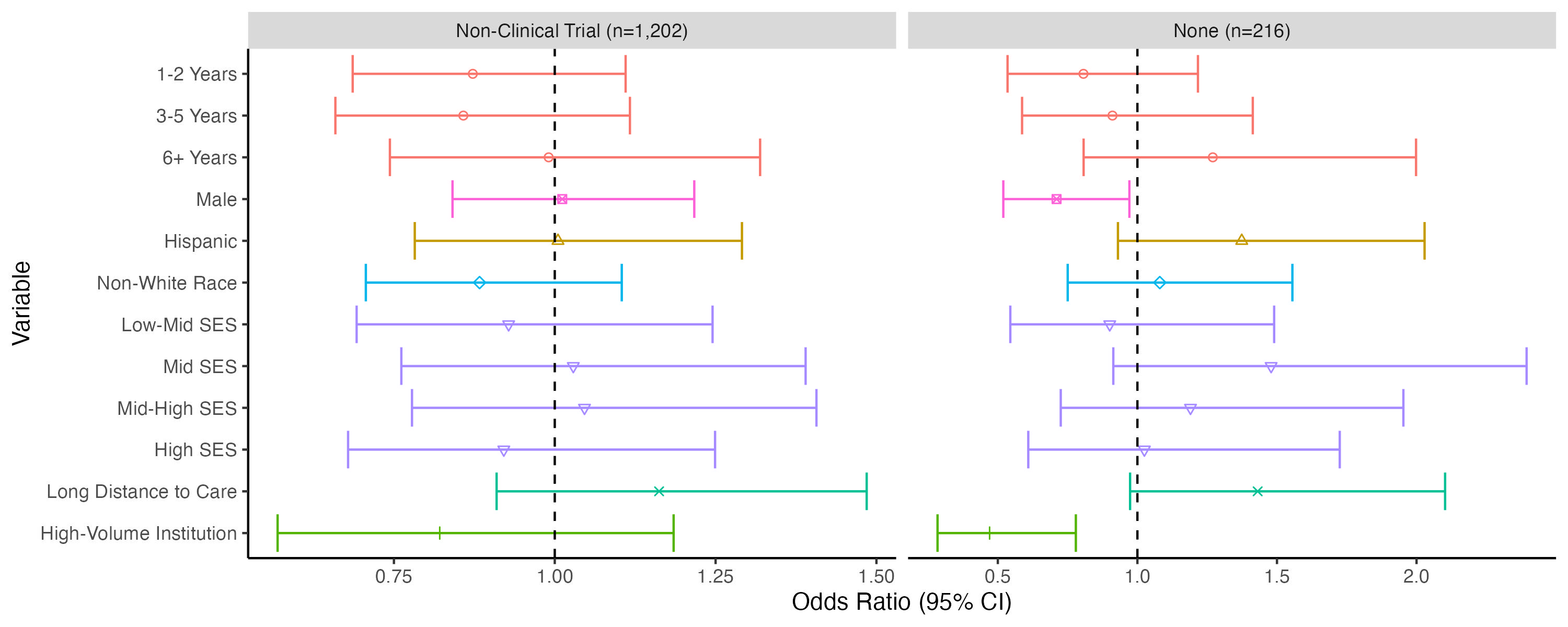

Supplement: Supplementary file 2 — Figure S2. [file CAM4-14-e70692-s001.jpeg]

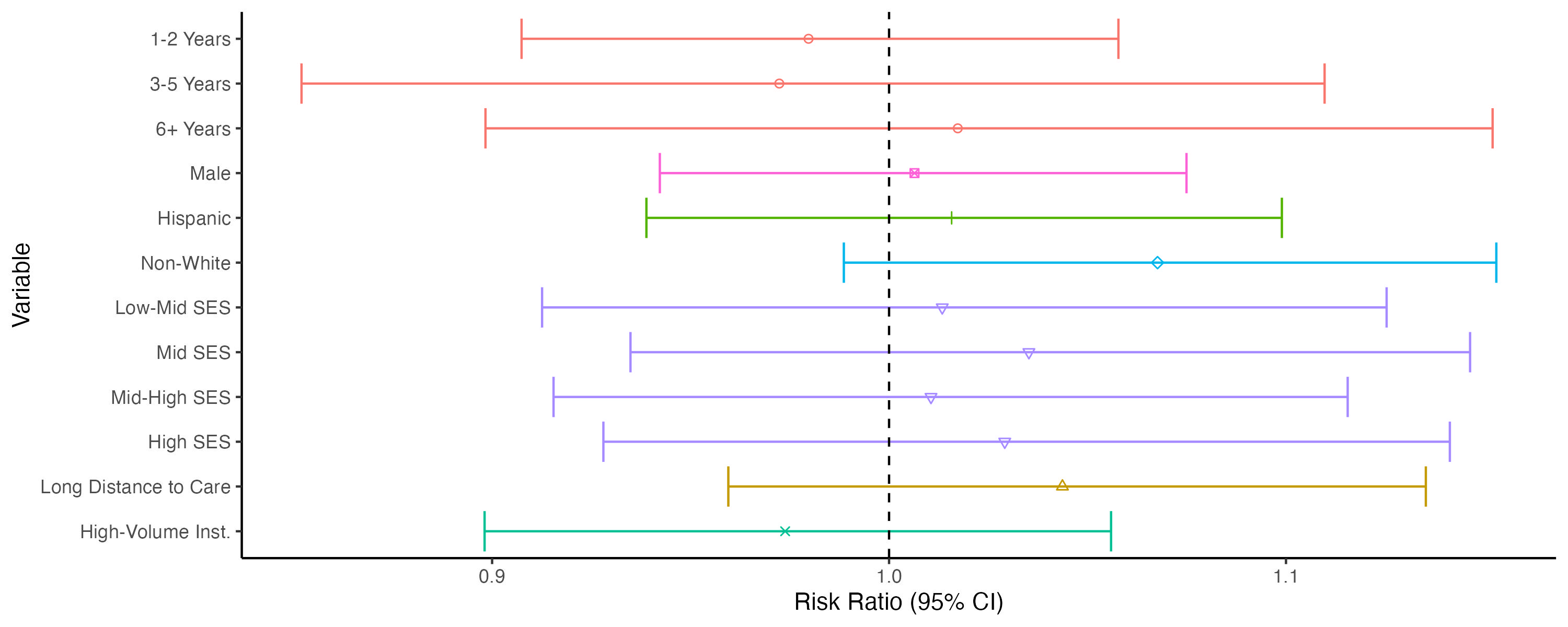

Supplement: Supplementary file 3 — Figure S3. [file CAM4-14-e70692-s003.jpeg]

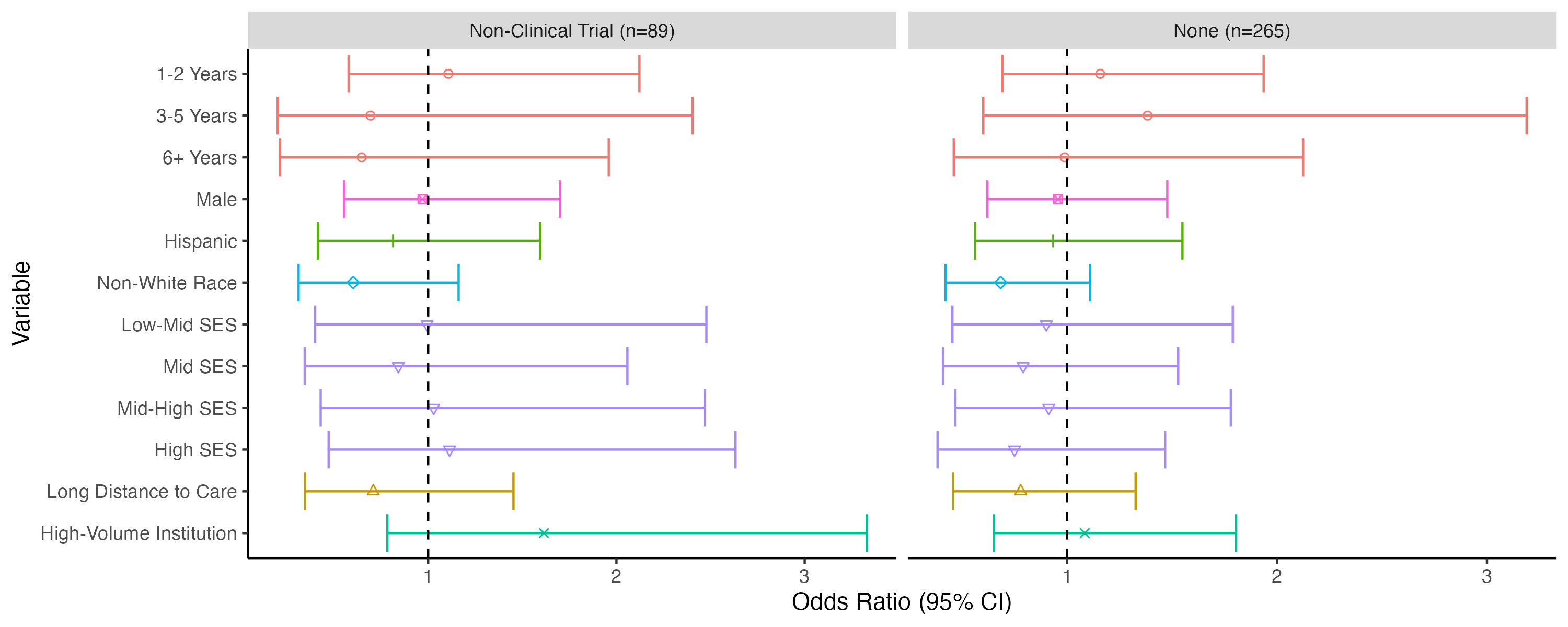

Supplement: Supplementary file 4 — Figure S4. [file CAM4-14-e70692-s004.jpeg]
