# Supplementary material for: Patterns of Clinical Trial Enrollment for Pediatric Patients With Hepatoblastoma and Wilms Tumor: A Report From the Children's Oncology Group
Source: Cancer Med. 2025 Mar 27;14(7):e70692. doi: 10.1002/cam4.70692 (PMC11950632; doi:10.1002/cam4.70692)
Supplement: Supplementary file 5 — Table S1. [file CAM4-14-e70692-s002.docx]

**Supplementary Materials**

**Supplementary Table 1. Description of non-therapeutic trials**

| **Protocol** | **Title** | **Open Periods** | **Inclusion Criteria** | **Exclusion Criteria** |
| --- | --- | --- | --- | --- |
| ACCL0431 | Effects of sodium thiosulfate versus observation on development of cisplatin-induced hearing loss in children with cancer | 2007-05 to 2015-01 | Aged 1-18 years; Planning to undergo treatment with any cisplatin-containing therapeutic regimen for cancer | No prior cisplatin |
| ACCL05C1 | Group-Wide, Prospective Study of Ototoxicity Assessment in Children Receiving Cisplatin Chemotherapy | 2007-05 to 2012-02 | Aged 1-30 years; Planning to undergo treatment with any cisplatin-containing therapeutic regimen for cancer; Intent to enroll on ACCL0431 | No prior cisplatin |
| ABTR01B1 | Collecting and Storing Malignant, Borderline Malignant Neoplasms, and Related Samples From Young Patients With Cancer | 2003-10 to 2018-06 | Aged 0-30 years; Diagnosed with primary neoplasm OR developed a second malignant neoplasm OR any diagnoses having an ICD-O Morphology Code ending in 1,2, or 3; Has biological specimens including solid tumors and leukemia available; Not eligible for disease-specific biology or banking protocol |  |
| ANBL00B1 | Biomarkers in Tumor Tissue Samples From Patients With Newly Diagnosed Neuroblastoma or Ganglioneuroblastoma | 2000-11 to 2100-01 | All newly diagnosed patients with suspected neuroblastoma, suspected ganglioneuroblastoma, or suspected ganglioneuroma/maturing subtype seen at Children's Oncology Group (COG) institutions; No prior chemotherapy with exception; A good faith effort to submit a neuroblastoma sample of sufficient quality for MYCN analysis | Patients with relapsed neuroblastoma who were not enrolled on ANBL00B1 at original diagnosis |
| ACCL1032 | Acupressure in Controlling Nausea in Young Patients Receiving Highly Emetogenic Chemotherapy | 2011-05 to 2016-05 | Aged 4-18 years; Newly diagnosed (i.e., not relapsed) with any malignancy; Current chemotherapy treatment plan must include at least 1 course of cisplatin at ≥ 50 mg/m2/dose or ifosfamide plus etoposide or doxorubicin or cyclophosphamide plus an anthracycline; The patient's current treatment plan must include an anti-emetic regimen with either ondansetron or granisetron on a scheduled basis | Prior history of acupressure use; Scheduled use of antiemetic agents other than ondansetron, granisetron, dexamethasone or aprepitant. Patients may receive other antiemetic agents PRN for breakthrough nausea/vomiting but not on a scheduled basis |
| COG 9346 | Hepatoblastoma Biology Study and Tissue Bank | 2005-09 to 2015-0 | Aged 6 months to 21 years; Patients with hepatoblastoma |  |
| ABTR04B1 | Collecting and Storing Tissue From Young Patients With Cancer | 2007-03 to 2024-12 | Aged 0-21 years; Any malignant tissue; Enrolled on a COG therapeutic, biology, or tissue banking protocol that allows collection of tissue for research and submission to a COG-designated resource laboratory; Patients with diagnosis pending are eligible |  |
| AREN03B2 | Study of Kidney Tumors in Younger Patients | 2006-02 to 2100-01 | Aged 0-29 years; Patients with the first occurrence of any tumor of the kidney identified on CT scan or MRI are eligible for this study; histologic diagnosis is not required prior to enrollment but is required for all patients once on study |  |
| APEC1621SC | Targeted Therapy Directed by Genetic Testing in Treating Pediatric Patients With Relapsed or Refractory Advanced Solid Tumors, Non-Hodgkin Lymphomas, or Histiocytic Disorders (The Pediatric MATCH Screening Trial) | 2017-07 to 2027-09 | Aged 12 months to 21 years; Patients with recurrent or refractory solid tumors, including non-Hodgkin lymphomas, histiocytoses (e.g. langerhans cell histiocytosis [LCH], juvenile xanthogranuloma [JXG], histiocytic sarcoma), and central nervous system (CNS) tumors are eligible; patients must have had histologic verification of malignancy at original diagnosis or relapse except in patients with intrinsic brain stem tumors, optic pathway gliomas, or patients with pineal tumors and elevations of cerebrospinal fluid (CSF) or serum tumor markers including alpha-fetoprotein or beta-human chorionic gonadotropin (HCG) | In cases where patient enrolls prior to histologic confirmation of recurrent disease, patient is ineligible and should be withdrawn from study if histology fails to confirm recurrence; please note: Patients with Hodgkin lymphoma and plexiform neurofibroma are not eligible |
| APEC14B1 | Project: Every Child for Younger Patients With Cancer | 2015-11 to 2030-12 | Aged 0-25 years; Enrollment must occur within 6 months of initial disease presentation OR within 6 months of refractory disease, disease progression, disease recurrence, second or secondary malignancy, or post-mortem; Patients previously enrolled on ACCRN07 are eligible to enroll on Tracking Outcome, Registry and Future Contact components of APEC14B1 any time after they reach age of majority; Patients with a known or suspected neoplasm that occurs in the pediatric, adolescent or young adult populations are eligible for enrollment |  |
| ACCL0934 | Levofloxacin in Preventing Infection in Young Patients With Acute Leukemia Receiving Chemotherapy or Undergoing Stem Cell Transplantation | 2011-06 to 2018-06 | 6 months to 21 years; Patient must fit 1 of the following 2 categories: Chemotherapy patients planned to receive at least 2 consecutive cycles (not required to be the first 2 cycles) of intensive chemotherapy OR Stem cell transplantation patients planned to receive at least 1 myeloablative autologous or allogeneic HSCT; Creatinine clearance or radioisotope glomerular filtration rate (GFR) > 70 mL/min/1.73 m^2 OR serum creatinine based on age/gender specifications | Patients previously enrolled on the trial are not eligible; therefore, patients with AL who were on study during intensive chemotherapy are not eligible to be enrolled during the HSCT; Patients with an allergy to quinolones; Patients with chronic active arthritis; Patients with a known pathologic prolongation of the corrected QT (QTc); Females who are pregnant or breast feeding; Patients being treated with antibacterial agents, with exception; Patients currently enrolled on the ACCL1034 study are not eligible until they have completed the 90 day observation period of that study |
| ACCL0731 | Glutamic Acid in Reducing Nerve Damage Caused by Vincristine in Young Patients With Cancer | 2006-08 to 2014-03 | Aged 3-21 years; Patients newly diagnosed with Wilm's tumor and scheduled to receive at least 9 consecutive weeks of chemotherapy with a vincristine-containing regimen; Patients newly diagnosed with rhabdomyosarcoma and scheduled to receive at least 9 consecutive weeks of chemotherapy with a vincristine-containing regimen; Patients newly diagnosed with ALL and scheduled to receive 4 consecutive weeks of chemotherapy with a vincristine-containing regimen with accompanying steroid therapy; Patients newly diagnosed with Non- Hodgkins Lymphoma (NHL) and scheduled to receive 4 consecutive weeks of chemotherapy with a vincristine-containing regimen with accompanying steroid therapy; Patients with no underlying neuromuscular disease or peripheral neuropathy | Abnormal baseline peripheral neurologic exam (i.e. or peripheral neuropathy); Patients with: seizure disorders  primary intracranial malignancy, family history of Charcot Marie Tooth Disease, a recent history of GuillianBarré26; Patients receiving concomitant itraconazole are at risk for increased vincristine toxicity and therefore are ineligible; Patients who are regularly using laxatives or stool softeners for constipation at the time of enrollment are not eligible to participate in the study. Likewise, since prevention of neuro-constipation will be evaluated, patients with an ongoing history of constipation that has required frequent use of laxatives or stool softeners should not be enrolled; Patients should not be scheduled to receive laxatives or stool softeners prophylactically to prevent constipation, as the prevention of neuro-constipation will be evaluated in this study; however, when patients show signs of developing constipation while on chemotherapy, as determined by the treating physician, they may be treated with laxatives or stool softeners at the clinician's discretion. Use of laxatives or stool softeners will be documented on the concomitant medication log |
| ADVL06B1 | Evaluating Dactinomycin and Vincristine in Young Patients With Cancer | 2008-02 to 2016-05 | Aged 0-16 years; Diagnosis of cancer; Due to receive or receiving dactinomycin and/or vincristine as a component of cancer treatment on another clinical trial; Able to comply with study requirements |  |

**Supplementary Table 2.** Demographic characteristics of Wilms tumor patients during the period where any strata were open.

|  | | **Trial Enrollment** | | |  |
| --- | --- | --- | --- | --- | --- |
| **Characteristic** | **N** | **Clinical Trial, N = 739^1^** | **Non-Therapeutic Study, N = 1,202^1^** | **None, N = 216^1^** | **Overall, N = 2,157^1^** |
| Sex | 2,157 |  |  |  |  |
| Female |  | 392 (53%) | 632 (53%) | 133 (62%) | 1,157 (54%) |
| Male |  | 347 (47%) | 570 (47%) | 83 (38%) | 1,000 (46%) |
| Race | 2,157 |  |  |  |  |
| White |  | 549 (74%) | 922 (77%) | 160 (74%) | 1,631 (76%) |
| Black |  | 134 (18%) | 196 (16%) | 40 (19%) | 370 (17%) |
| Native American/Alaska Native |  | 5 (0.7%) | 7 (0.6%) | 0 (0%) | 12 (0.6%) |
| Asian/PI |  | 12 (1.6%) | 24 (2.0%) | 4 (1.9%) | 40 (1.9%) |
| Other |  | 39 (5.3%) | 53 (4.4%) | 12 (5.6%) | 104 (4.8%) |
| Hispanic, Yes | 2,157 | 122 (17%) | 201 (17%) | 45 (21%) | 368 (17%) |
| Age at Diagnosis (Years) | 2,157 |  |  |  |  |
| <1 |  | 183 (25%) | 323 (27%) | 55 (25%) | 561 (26%) |
| 1-2 |  | 255 (35%) | 393 (33%) | 63 (29%) | 711 (33%) |
| 3-5 |  | 177 (24%) | 269 (22%) | 50 (23%) | 496 (23%) |
| 6+ |  | 124 (17%) | 217 (18%) | 48 (22%) | 389 (18%) |
| Year of Diagnosis | 2,157 |  |  |  |  |
| 2007 |  | 4 (0.5%) | 1 (<0.1%) | 4 (1.9%) | 9 (0.4%) |
| 2008 |  | 83 (11%) | 111 (9.2%) | 13 (6.0%) | 207 (9.6%) |
| 2009 |  | 128 (17%) | 149 (12%) | 31 (14%) | 308 (14%) |
| 2010 |  | 125 (17%) | 142 (12%) | 25 (12%) | 292 (14%) |
| 2011 |  | 152 (21%) | 126 (10%) | 18 (8.3%) | 296 (14%) |
| 2012 |  | 154 (21%) | 105 (8.7%) | 19 (8.8%) | 278 (13%) |
| 2013 |  | 65 (8.8%) | 153 (13%) | 20 (9.3%) | 238 (11%) |
| 2014 |  | 26 (3.5%) | 193 (16%) | 30 (14%) | 249 (12%) |
| 2015 |  | 2 (0.3%) | 180 (15%) | 35 (16%) | 217 (10%) |
| 2016 |  | 0 (0%) | 42 (3.5%) | 21 (9.7%) | 63 (2.9%) |
| Yost Index (Quintiles) | 2,157 |  |  |  |  |
| Low SES |  | 151 (20%) | 245 (20%) | 42 (19%) | 438 (20%) |
| Low-Mid SES |  | 149 (20%) | 232 (19%) | 38 (18%) | 419 (19%) |
| Mid SES |  | 138 (19%) | 236 (20%) | 53 (25%) | 427 (20%) |
| Mid-High SES |  | 149 (20%) | 259 (22%) | 45 (21%) | 453 (21%) |
| High SES |  | 152 (21%) | 230 (19%) | 38 (18%) | 420 (19%) |
| Distance to Care, per 50 km | 2,157 | 0.59 (0.28, 1.49) | 0.68 (0.29, 1.60) | 0.63 (0.27, 1.89) | 0.65 (0.28, 1.58) |
| High Volume Institution | 2,157 | 692 (94%) | 1,112 (93%) | 189 (88%) | 1,993 (92%) |
| ^1^n (%); Median (IQR) | | | | | |

**Supplemental Table 3.** Demographic characteristics of Hepatoblastoma patients during the period where any strata were open.

|  | | **Trial Enrollment** | | |  |
| --- | --- | --- | --- | --- | --- |
| **Characteristic** | **N** | **Clinical Trial, N = 135^1^** | **Non-Therapeutic Study, N = 89^1^** | **None, N = 265^1^** | **Overall, N = 489^1^** |
| Sex | 489 |  |  |  |  |
| Female |  | 50 (37%) | 34 (38%) | 101 (38%) | 185 (38%) |
| Male |  | 85 (63%) | 55 (62%) | 164 (62%) | 304 (62%) |
| Race | 489 |  |  |  |  |
| White |  | 95 (70%) | 71 (80%) | 204 (77%) | 370 (76%) |
| Black |  | 16 (12%) | 9 (10%) | 30 (11%) | 55 (11%) |
| Native American/Alaska Native |  | 0 (0%) | 1 (1.1%) | 3 (1.1%) | 4 (0.8%) |
| Asian/PI |  | 13 (9.6%) | 4 (4.5%) | 15 (5.7%) | 32 (6.5%) |
| Other |  | 11 (8.1%) | 4 (4.5%) | 13 (4.9%) | 28 (5.7%) |
| Hispanic, Yes | 489 | 33 (24%) | 19 (21%) | 64 (24%) | 116 (24%) |
| Age at Diagnosis (Years) | 489 |  |  |  |  |
| <1 |  | 84 (62%) | 57 (64%) | 156 (59%) | 297 (61%) |
| 1-2 |  | 30 (22%) | 23 (26%) | 66 (25%) | 119 (24%) |
| 3-5 |  | 9 (6.7%) | 4 (4.5%) | 22 (8.3%) | 35 (7.2%) |
| 6+ |  | 12 (8.9%) | 5 (5.6%) | 21 (7.9%) | 38 (7.8%) |
| Year of Diagnosis | 489 |  |  |  |  |
| 2009 |  | 9 (6.7%) | 10 (11%) | 4 (1.5%) | 23 (4.7%) |
| 2010 |  | 51 (38%) | 6 (6.7%) | 35 (13%) | 92 (19%) |
| 2011 |  | 39 (29%) | 8 (9.0%) | 31 (12%) | 78 (16%) |
| 2012 |  | 16 (12%) | 20 (22%) | 45 (17%) | 81 (17%) |
| 2013 |  | 9 (6.7%) | 14 (16%) | 41 (15%) | 64 (13%) |
| 2014 |  | 7 (5.2%) | 11 (12%) | 39 (15%) | 57 (12%) |
| 2015 |  | 3 (2.2%) | 16 (18%) | 42 (16%) | 61 (12%) |
| 2016 |  | 1 (0.7%) | 4 (4.5%) | 23 (8.7%) | 28 (5.7%) |
| 2017 |  | 0 (0%) | 0 (0%) | 5 (1.9%) | 5 (1.0%) |
| Yost Index (Quintiles) | 489 |  |  |  |  |
| Low SES |  | 29 (21%) | 17 (19%) | 61 (23%) | 107 (22%) |
| Low-Mid SES |  | 22 (16%) | 14 (16%) | 44 (17%) | 80 (16%) |
| Mid SES |  | 28 (21%) | 15 (17%) | 50 (19%) | 93 (19%) |
| Mid-High SES |  | 28 (21%) | 21 (24%) | 61 (23%) | 110 (22%) |
| High SES |  | 28 (21%) | 22 (25%) | 49 (18%) | 99 (20%) |
| Distance to Care, per 50 km | 489 | 0.86 (0.39, 1.86) | 0.63 (0.35, 1.37) | 0.68 (0.31, 1.65) | 0.69 (0.33, 1.65) |
| High Volume Institution | 489 | 105 (78%) | 76 (85%) | 210 (79%) | 391 (80%) |
| ^1^n (%); Median (IQR) | | | | | |
